# Cancer diagnostic assessment programs: standards for the organization of care in Ontario

**DOI:** 10.3747/co.v16i6.400

**Published:** 2009-12

**Authors:** M. Brouwers, T.K. Oliver, J. Crawford, P. Ellison, W.K. Evans, A. Gagliardi, J. Lacourciere, D. Lo, V. Mai, S. McNair, T. Minuk, L. Rabeneck, C. Rand, J. Ross, J. Smylie, J. Srigley, H. Stern, M. Trudeau

**Affiliations:** * Program in Evidence-Based Care, Hamilton, ON; † Department of Clinical Epidemiology and Biostatistics, McMaster University, Hamilton, ON; ‡ University Health Network, Toronto, ON; § Juravinski Cancer Centre, Hamilton, ON; || Toronto General Research Institute, Toronto, ON; # Thunder Bay Regional Health Sciences Centre, Thunder Bay, ON; ** Department of Medical Oncology, University of Toronto, Toronto, ON; †† Cancer Care Ontario, Toronto, ON; ‡‡ Department of Radiology, Henderson Hospital, Hamilton, ON; §§ Ottawa Regional Cancer Centre, Ottawa, ON; |||| Jewish General Hospital, Montreal, QC

**Keywords:** Diagnostic assessment, organizational, standards, systematic review, cancer

## Abstract

**Background:**

Improving access to better, more efficient, and rapid cancer diagnosis is a necessary component of a high-quality cancer system. How diagnostic services ought to be organized, structured, and evaluated is less understood and studied. Our objective was to address this gap.

**Methods:**

As a quality initiative of Cancer Care Ontario’s Program in Evidence-Based Care, the Diagnostic Assessment Standards Panel, with representation from clinical oncology experts, institutional and clinical administrative leaders, health service researchers, and methodologists, conducted a systematic review and a targeted environmental scan of the unpublished literature. Standards were developed based on expert consensus opinion informed by the identified evidence. Through external review, clinicians and administrators across Ontario were given the opportunity to provide feedback.

**Results:**

The body of evidence consists of thirty-five published studies and fifteen unpublished guidance documents. The evidence and consensus opinion consistently favoured an organized, centralized system with multidisciplinary team membership as the optimal approach for the delivery of diagnostic cancer assessment services. Independent external stakeholders agreed (with higher mean values, maximum 5, indicating stronger agreement) that dap standards are needed (mean: 4.6), that standards should be formally approved (mean: 4.3), and importantly, that standards reflect an effective approach that will lead to quality improvements in the cancer system (mean: 4.5) and in patient care (mean: 4.3).

**Interpretation:**

Based on the best available evidence, standards for the organization of daps are offered. There is clear need to integrate formal and comprehensive evaluation strategies with the implementation of the standards to advance this field.

## 1. INTRODUCTION

The provision of efficient and rapid cancer diagnosis is a necessary component of a high-quality cancer system, but how diagnostic services ought to be organized, structured, and evaluated is less understood and studied. The inefficient and inappropriate use of diagnostic imaging procedures (test duplication, inappropriate tests ordered) can have substantial resource implications and can delay patient treatment—a serious health care concern. One prospective Canadian study found that median wait times to diagnosis were 37 days, 71 days, and 81 days for patients with lung, colorectal, and prostate cancer respectively 1. In relation to lung cancer, Liberman *et al.* 2 reported mean and median wait times of 208 and 109 days respectively between initial contact with a physician or first onset of symptoms and diagnostic surgery. Similarly, data from seven Canadian provinces measuring the time from an abnormal breast screen to diagnosis showed a median time to diagnosis of 3.7 weeks; 10% of women waited 9.6 weeks or longer for a diagnosis 3.

Diagnostic assessment programs (daps) are one component of an overall rapid-access strategy for diagnosis. The daps may be either actual or virtual entities characterized by facilitated access to comprehensive diagnostic services, multidisciplinary consultative expertise, patient information resources, and psychosocial supports. Programs of this type have been associated with high patient satisfaction 4–7, a reduction in time from diagnosis to the initiation of treatment for various disease sites 5,8, and potentially, improvements in clinical outcomes 9. However, less clear are the organizational and practice setting features that define a high-quality dap, the role of a dap in a comprehensive rapid-access strategy, the defining features of a dap that lend themselves to unique geographic or jurisdictional situations, and the indicators that should be used to measure quality and impact.

In the province of Ontario, the population of approximately 12 million people is spread over more than 1 million square kilometres, and the distribution of new cancer cases varies considerably across the various regions serving that population 10. Population size and geographic spread are important considerations in strategizing about quality improvement actions meant to increase access and to reduce wait times to diagnosis. At the same time, it must be acknowledged that solutions for one region may or may not be generalizable to another. In Ontario, approximately 65,000 new cases of cancer per year are predicted 11, with most patients presenting with lung, breast, colorectal, or prostate cancer 11. These patients will require a high standard of care, starting with their entry into the cancer system. As opposed to current opportunistic systems, an organized entry into the cancer system and diagnostic processes has the potential to reduce duplication of tests, to improve efficiency, to reduce costs and waiting times, to enhance the overall quality of care for patients throughout the cancer system, and conceivably, to improve the outcome of treatment.

The objectives of the Ontario standards for the organization of care for cancer daps are to provide advice to administrators, planners, and government on the optimal strategic planning and investment options required to provide the highest standard of care for patients with cancer. The Diagnostic Assessment Standards Panel was convened to work with the Program in Evidence-Based Care (pebc) to develop recommendations that could guide the design, implementation, and evaluation of daps in Ontario.

## 2. METHODS

The Diagnostic Assessment Standards Panel, composed of clinical oncology experts, regional vice presidents, clinical administrative leaders, health service researchers, and methodologists (Table I), conducted a systematic review and environmental scan of the literature to help inform the development of provincial standards. External validation of the standards was conducted through an external review by relevant practitioners and administrators throughout the province of Ontario.

### 2.1 Search Strategy

A systematic review published by Gagliardi *et al.* in 2004 12 served as the evidence foundation for the current standards for practice. In that systematic review, the authors identified twenty relevant studies, published up to 2002, that evaluated both the clinical and the economic components of daps for suspected cases of breast, colorectal, lung, head-and-neck, prostate, and other cancers.

The search of the literature was conducted using mesh and the keyword terms “ambulatory care facilities/ OR community health centers/ OR outpatient clinics, OR hospital/Ambulatory Care/ OR cancer care facilities/ OR (keywords: rapid or same day or one stop or multidisciplinary AND clinic AND diagnosis) AND Breast Neoplasms/di OR Prostatic neoplasms/di OR Lung neoplasms/di OR Exp colorectal neoplasms/di or Exp head and neck neoplasms/di OR prostatic neoplasms OR breast neoplasms OR lung neoplasms OR exp colorectal neoplasms OR Exp head and neck neoplasms”. The search was limited to English-language citations.

The original literature search, which spanned 1985–2002, was updated to October 2006 using medline (ovid: 2002 through October 2006), embase (ovid: 2002 to October 2006), the Cochrane Library (ovid; Issue 3, 2006), the Canadian Medical Association Infobase, and the National Guideline Clearinghouse. Reference lists of related papers and recent review articles were also scanned for additional citations.

### 2.2 Selection Criteria

Articles were included in the systematic review of the evidence they met these criteria:

- ***Study design:***Randomized controlled trials (rcts), case–control studies, and prospective or retrospective cohort studies (letters, editorials, and comments were excluded)
- ***Publication interval:***January 2002 through October 2006
- ***Unit of study:***
  - Diagnostic assessment programs or units, or one-stop, fast-track, or rapid-access clinics with
  - a focus on care provision for patients with suspected cancer, and
  - encompassing the diagnostic assessment of patients with a potential malignancy.
- ***Language:***English

Quality of the primary studies was assessed using common appraisal tools, including the domains from the Jadad scale 13 (for rcts) and Downs and Black 14 for observational studies.

The environmental scan involved two processes. First, inquiries were made directly to key cancer leaders and contacts in Ontario, Canada, and to selected groups outside of Canada. Second, a targeted Internet search was undertaken of key sites, including professional associations, guideline registries, and health care organizations (Table II). Any reports detailing models, frameworks, descriptions, and evaluations of daps (including quality improvement initiatives) from these targeted individuals, organizations, or information sources were considered eligible for inclusion. No specific quality evaluation criteria were applied, because no scales or quality domains have been evaluated using traditional health measurement principles.

## 3. RESULTS

The evidence base comprises thirty-four published studies 4,7,15–46 and fifteen unpublished guidance documents 47–61. The present report focuses on a brief overview of the evidence found. The reader is referred to the full systematic review and environmental scan report (published elsewhere) for a full description and report of the evidence 62.

### 3.1 Systematic Review

#### 3.1.1 Search Results

The original systematic review by Gagliardi *et al.* 12 included twenty articles that described outcomes related to specific disease-site assessment units: eleven for breast cancer 4,15–24, three for colorectal cancer 7,25,26, and six for head-and-neck cancer 31–36. There were seventeen case series that involved 38–3119 patients, two rcts that included 478 and 791 patients, and one case–control study that included 177 cases and 162 controls 4,7,15–26,31–36. The update of the literature search identified 823 citations in which patient outcomes related to diagnostic assessment units were described for colorectal cancer in four studies 27–30, head-and-neck cancer in two studies 37,38, lung cancer in two studies 39,40, gynecologic cancers in three studies 41–43, neurologic cancers in one study 44, lymph node cancers in one study 45, and upper gastrointestinal cancers in one study 46. Study designs included one small rct (88 patients), seven prospective cohort studies (359–3637 patients), and six retrospective studies (69–930 patients) 27–30,37–46.

Elements of the Downs and Black quality assessment scale for observational studies 14 were used to assess the quality of relevant studies included in the updated review. Four key domains were used in the evaluation of comparability of subjects, exposure or intervention, outcome measure, and statistical analysis. The quality of the studies was variable, but generally modest, with approximately half the studies not using a comparative control group, thus increasing the risk for selection bias.

#### 3.1.2 Outcomes

The overall findings from Gagliardi *et al.* 12 included the benefits of diagnostic assessment services in terms of reduced wait times for specific diagnostic procedures, increased patient satisfaction, and reduced anxiety for patients with negative findings. Most patients were diagnosed at the initial visit, and most diagnoses were confirmed by a pathology determination. A number of studies reported increased anxiety in women diagnosed with breast cancer at one-stop clinics, and one study measured clinical outcomes for breast cancer patients.

For the updated systematic review, all studies but one were undertaken in the United Kingdom and included the National Health Service referral guidelines as a quality performance indicator for improving timely access 27–30,37–41,43–46. Only one study evaluated the cost of follow-up visits to general practitioners in an rct evaluating a centralized two-stop rapid assessment unit against conventional routine diagnostic evaluation 39. Ten studies defined cancer-specific risk criteria for general practitioners to utilize in their risk assessment and decision-making to expedite high-risk referrals to rapid diagnostic units 27,28,30,38,41–46. Numerous studies evaluated or addressed a multidisciplinary team approach for the rapid diagnostic assessment of cancer 37–40,43,45.

The findings from the update of the literature were similar to those reported by Gagliardi *et al.* 12:

- Most of the studies evaluating rapid diagnostic assessment for suspected cases of cancer demonstrated a reduced time from first referral to specialist visit and time to first treatment in that setting.
- The studies that evaluated patient satisfaction found greater patient satisfaction with service provision and personal care given by medical staff 30,35,42.
- Studies assessing multidisciplinary care found that it translated into a more comprehensive patient assessment and might contribute to better care overall 35,37,38,40,43,45,.
- Various studies reported that specific referral criteria for individual cancer types aided in decision-making for general practitioners and might assist in ensuring appropriate referral for high-risk suspected cases of cancer to rapid daps 27,28,30,38,41–46.

### 3.2 Environmental Scan

#### 3.2.1 Search Results

The environmental scan found fifteen guidance documents on the organization of cancer diagnostic services. Although it was not the specific stated purpose of many of the documents, some organizational elements of daps were addressed in each of the guidance documents—for example, mandate, centralized access, scope of diagnostic activity, team criteria, linkages and collaborations, volume prerequisites, and quality indicators. In most cases, the conclusions derived from the guidance documents were supported by consensus-level evidence.

#### 3.2.2 Outcomes

A consistent message was that coordinated and organized diagnostic assessment services managed by multidisciplinary teams with operational links to other specialty services resulted in reduced wait times and improved services—and possibly in improved patient outcomes. The guidance documents also outlined many of the requirements for a dap, including centralized access to diagnostic assessment services, multidisciplinary team criteria, and the diagnostic services needed to successfully operate a dap. Centralized access was most commonly characterized as a one-stop clinic, with integrated and coordinated cancer services, that provides seamless diagnostic assessment services.

The composition of the disease-specific multidisciplinary team included not only the appropriate spectrum of disease-specific professionals needed to perform a diagnostic assessment, along with the appropriate disease-specific support personnel, but also coordinators and directors or chairs who were recommended to ensure the coordination of services.

The common clinical examination, imaging, diagnostic, and staging procedures and surgical consultation procedures were listed in the guidance documents. Also reported were the pathology services, disease-specific tests, and supportive services that might be needed as part of the spectrum of diagnostic care. There was a general indication in the documents that the appropriate diagnostic investigations and procedures would lead to improved services and patient outcomes.

Several of the guidance documents reported the need for linkages to maintain communication between primary health care providers and the coordinated diagnostic and treatment services as patients navigate the system. It was suggested that, in low-volume or underserviced areas, smaller programs should have formal collaborative links with larger programs.

There was little evidence to indicate the patient volumes required to maintain one-stop daps. Each jurisdiction would need to determine the appropriate volume requirements for each type or model of dap implemented.

Several documents established indicators of quality, with wait times being the most common indicator reported. Other documents recommended that the time from signs or symptoms suggestive of cancer to diagnosis should not exceed 4 weeks. A more thorough analysis of benchmarking is warranted. The development of quality assurance through performance measurement and audit programs was also recommended.

## 4. CONSENSUS PROCESS AND EXTERNAL REVIEW

The Diagnostic Assessment Standards Panel used the evidence that was available from the published literature, the environmental scan, and their expert opinion to reach consensus for standards on the organization and delivery of diagnostic assessment services in Ontario. The process of developing standards included the formation of the Diagnostic Assessment Standards Panel with a subset working group responsible for writing the draft standards. The panel met often through teleconferences and once in person to draft and approve the standards for practice before the standards were sent for external review. Approval was obtained through informal consensus at the meetings and also through an e-mail survey with 10 questions asking about the level of agreement with the completeness of the evidentiary base and the recommendations as stated. Conflicting views were noted and discussed, and it was agreed that the majority opinion of the panel would be adopted.

Upon final approval of the draft by the Diagnostic Assessment Standards Panel, the document underwent internal review by the Report Approval Panel and the Scientific Manager of the pebc. The draft standards were then distributed for review to 74 external Ontario stakeholders: 24 primary care providers, 17 chairs of provincial disease site groups, 25 regional vice presidents of cancer programs and senior administrators, and 8 cancer screening program experts. External review included the opportunity for written feedback and a survey on level of agreement with the manner of evidence collection, with the process used to derive recommendations, and with the recommendations themselves. Responses were received from 11, 3, 12, and 5 participants in each of the respective groups (41% overall return rate). The written feedback from both the clinical and the administrative experts was similar in nature.

Feedback was extremely positive. Most stakeholders agreed (with higher mean values, maximum 5, indicating stronger agreement) that there was a need for dap standards (mean: 4.6), that the standards were clear (mean: 4.1), that the draft standards as stated were acceptable (mean: 4.2), that the standards should be formally approved (mean: 4.3), and importantly, that the standards reflect an effective approach that will lead to quality improvements in the cancer system (mean: 4.5). There was also some indication that the standards would be challenging to implement (mean: 3.9), but that the draft standards for the organization of care were achievable (mean: 4.0) and would reflect a more desirable system than current practice for improving the quality of patient care (mean: 4.3).

No major modifications to the draft standards were deemed necessary after external review; however, several minor modifications that had been suggested were discussed and incorporated into the draft. Upon final review, the standards were presented to the Executive Team and the Board of Cancer Care Ontario, and the final version of the standards was formally approved by the Diagnostic Assessment Standards Panel. The final approved standards are set out in Appendix A.

## 5. CONCLUSIONS

It is clear that organized, centralized systems with multidisciplinary team membership are considered the optimal organization for the delivery of diagnostic cancer assessment services. Even though much of the available literature is limited in quality, and expert consensus opinion was often used to inform the guidance documents, the evidence across studies, the statements of credible guidance organizations, and the expert consensus opinion of the Diagnostic Assessment Standards Panel all deliver a consistent message.

There are, however, significant and frequently cited challenges associated with the implementation of dap programs. There is a general consensus that implementation of the standards would not be cost-neutral and that additional resources (that is, human resources, new equipment, equipment replacement, and appropriate fees and incentives) would likely be necessary. The reallocation of scarce resources would likely cause hardship on other components of the cancer system, not only in terms of cost, but also in terms of demand for services beyond diagnostic assessment—that is, moving patients at a faster rate into treatment, with the associated potential for backlogging at that juncture. The transition protocol between diagnostic assessment and treatment management with multidisciplinary team membership would need to be carefully mapped out according to service and jurisdictional demands. The reorganization of care would also require strong and collaborative leadership between clinicians, clinical administrators, hospital ceos, it leaders, and the local health integration networks across a variety of settings. The confluence between cancer and non-cancer diagnostic care agendas was also seen as a barrier to implementation. The ability to affect change is limited in a system defined by multiple stakeholders representing many types of diseases, with cancer being only one; the competition with other non-cancer programs could create access barriers to clinicians and equipment. In addition, there may be challenges with the communication required to facilitate buy-in by all providers. There is also concern regarding the need for adequate it systems and connectivity, particularly in regions with a large rural demographic, where the “virtual program” model and single central registry are particularly relevant.

These are daunting challenges. However, success models emerging in Ontario show that the implementation of a dap can be achieved without undue burden to the health system. In Ottawa, a collaborative model of surgical cancer care was developed with the primary tertiary centre anchoring a virtual model with eight partnering hospitals in the region. An integral part of this model was the development of diagnostic assessment units (for patients with thoracic cancer, colorectal cancer, breast cancer, and prostate cancer) that have been opened under the umbrella of a central cancer assessment clinic. The cancer assessment clinic was developed to act as a central access point offering coordinated and streamlined multidisciplinary care, where a patient with a suspicion of cancer enters a system (organized by the four disease sites) that acts as the gateway and triage centre for access to coordinated cancer services. Under this system, important collaborative linkages, known as “communities of practice,” have been established across the region, and improvements in patient and system outcomes, such as reductions in wait times, have been observed (Fung-Kee-Fung M. The Ottawa Hospital. Personal communication).

It is hoped that the organizational standards will be a useful tool in the development of diagnostic assessment models across various jurisdictions. It is also hoped that, regardless of the model chosen, coordinated rapid access to care in a multidisciplinary team environment will result in a “raising of the bar” in the provision of timely diagnostic assessment services to patients.

The standards concerning daps were generated to meet the demand of cancer diagnostic assessment services in Ontario, but the structure and organization of a dap will be influenced by the regional and geographic realities of each jurisdiction, the diagnostic tests necessary to assess an organ system (symptom complexity or physical abnormalities, for instance), and the anticipated volume of cases. Hence, it is reasonable to suggest that the standards will also be generalizable to other jurisdictions outside of Ontario.

Regardless of the dap structure implemented in any given jurisdiction, there will be an ongoing need for a comprehensive and formal evaluation strategy not only to refine existing and future diagnostic assessment services in Ontario, but also to help develop a more complete evidence base concerning the value of organized daps across many jurisdictions.

## 6. CONFLICT OF INTEREST

No conflicts of interest were declared. This project was sponsored by the Ontario Ministry of Health and Long-Term Care through Cancer Care Ontario’s Program in Evidence-Based Care.

## Figures and Tables

**TABLE I:** Membership of the Diagnostic Assessment Programs Standards Panel

Melissa Brouwers md (facilitator)	Director, pebc, cco, and Associate Professor (PT), Department of Clinical Epidemiology and Biostatistics, McMaster University, Hamilton, ON
Terry Minuk md (diagnostic radiology specialist)	Hamilton, ON
Joanne Crawford msc (nursing)	McMaster University, Hamilton, ON
Tom Oliver	Research Coordinator, pebc, cco, McMaster University, Hamilton, ON
Phil Elison md (family medicine)	Liaison from the Ontario College of Family Physicians to cco, Toronto, ON
Linda Rabeneck md (gastroenterologist)	Vice President, Regional Cancer Services, cco, Toronto, ON
William K. Evans md (medical oncologist, lung cancer specialist)	Co-Chair, Lung Disease Site Group, pebc, cco, and Vice-President, Regional Cancer Services, cco, Hamilton, ON
Carol Rand	Regional Director, Systemic, Supportive and Palliative Care, Juravinski Cancer Centre, Hamilton, ON
Anna Gagliardi phd	Scientist, Sunnybrook Research Institute, and Assistant Professor, Departments of Surgery and of Health Policy, Management and Evaluation, Faculty of Medicine, University of Toronto, Toronto, ON
Jill Ross	Director, Clinical Programs, cco, Toronto, ON
Joanne Lacourciere	Manager, Northwest Regional Cancer Program, Thunder Bay Regional Health Sciences Centre, Thunder Bay, ON
Jennifer Smylie	Clinical Manager, Regional Assessment Centre for Lung, Colorectal and Prostate Cancers, The Ottawa Hospital Regional Cancer Centre, Ottawa, ON
Dorothy Lo md (medical oncologist)	Medical oncology resident, University of Toronto, and Master of Health Sciences student, University of Toronto, Toronto, ON
John Srigley md (pathologist)	Provincial Head, Laboratory Medicine/Pathology, cco, Kingston, ON
Verna Mai md	Director, Screening Program, cco, Toronto, ON
Hartley Stern md (surgeon)	Provincial Head, Surgical Oncology, and Vice-President, Regional Cancer Services, cco, Ottawa, ON
Sheila McNair phd	Assistant Director, pebc, cco, McMaster University, Hamilton, ON
Maureen Trudeau md (medical oncologist, breast cancer specialist)	Co-Chair, Breast Disease Site Group, pebc, and Provincial Head, Systematic Therapy Program, cco, Toronto, ON

pebc = Program in Evidence-Based Care; cco = Cancer Care Ontario.

**TABLE II:** Environmental scan of the literature

Target	Source	Method
Local jurisdictions	Ontario regions	Direct inquiry
	British Columbia	Direct inquiry
	Alberta	Direct inquiry
	Saskatchewan	Direct inquiry
	Manitoba	Direct inquiry
	Quebec	Direct inquiry
	Nova Scotia	Direct inquiry
	Newfoundland	Direct inquiry
Guideline directories	Ontario Guidelines Advisory Committee	Internet search
Other	American Society of Clinical Oncology	Internet search
	American College of Radiologists	Direct inquiry, Internet search
	Canadian Association of Radiologists	Direct inquiry, Internet search
	Canadian Strategy for Cancer Control	Direct inquiry, Internet search
	National Health Services, United Kingdom	Internet search
	Scottish Intercollegiate Guidelines Network, Scotland	Internet search
	Standards, Options, Recommendations, France	Internet search
	Veterans Affairs, United States	Internet search
	New Zealand	Internet search
	Australia	Direct inquiry, Internet search

## APPENDIX A ONTARIO STANDARDS FOR DIAGNOSTIC ASSESSMENT PROGRAMS

The following standards for practice were informed by modest evidence from thirty-four published studies and fifteen unpublished guidance documents, but were primarily derived through the expert consensus opinion of the Diagnostic Assessment Programs Standards Panel. The standards were reviewed externally by Ontario stakeholders, including primary care providers, chairs of Ontario provincial disease site groups, regional vice presidents of cancer programs, senior administrators, and cancer screening program experts.

### SCOPE

Improving access to better and more rapid cancer diagnosis has been identified as a priority for Cancer Care Ontario (cco) and the Government of Ontario. A first step in realizing this objective is the development of provincial standards that define the organizational and practice-setting features expected of a diagnostic assessment program (dap). These standards represent one of a series of strategies that are needed to achieve the overall goal of improved rapid access to diagnosis. The standards that follow, which were developed by the Diagnostic Assessment Standards Panel, apply to the organization of daps and include the full spectrum of multidisciplinary diagnostic assessment leading to treatment. These standards will be routinely updated as the evidentiary support for the recommendations, particularly the evidence related to evaluation and outcomes data, matures.

### PURPOSE AND PRINCIPLES

The mandate of a dap is to coordinate patient care from referral to definitive diagnosis. These are the guiding principles for the dap:

- To ensure that an environment of patient-centred care is established
  - Patients have equal access to high-quality diagnostic care regardless of where they live in the province.
  - Patients are supported throughout the diagnostic process.
  - Patients have a diagnosis of cancer made or ruled out in a timely fashion.
- To ensure that a coordinated referral and follow-up system is established
- To ensure that indicators of quality are established and monitored to evaluate performance outcomes
- The objectives of the dap will be enabled by the development and implementation of common evidence-based regional or provincial guidelines (or both), which may include:
  - Disease-specific protocols regarding diagnostic work-ups
  - Service frameworks for primary care providers
  - Wait-time benchmarks

The dap must be able to demonstrate compliance (alignment) with these principles.

### DIAGNOSTIC ASSESSMENT PROGRAMS

The structure and organization of a dap will be influenced by the regional and geographic realities of each jurisdiction, the diagnostic tests necessary to assess an organ system (dealing with symptom complexity or physical abnormalities), and the anticipated volume of cases. Two core organizational models are recommended:

- One-Stop Diagnostic Assessment Services
  One-stop single-location assessment services are those that provide the totality of diagnostic services in one place and, where clinically appropriate, within one patient visit.
  - One-stop assessment services may also provide total service across the cancer continuum (that is, from screening to diagnosis to treatment and follow-up).
  - The size of the region and the scope of care provided (that is, diagnostic versus total care) will determine whether a region will have one or more programs.
  - For rare cancers or where diagnostic and treatment centres of excellence already exist, diagnostic assessment services in one region may also provide services to patients from several regions.
  - The organization of assessment services will typically be disease-site specific, but in some cases, an assessment program may oversee multiple tumour types.
- Virtual Diagnostic Assessment Services
  Where patient populations and geographic dispersion do not permit single-location assessment services, virtual programs should be explored
  - within a region or city.
    These virtual systems of diagnostic services are spread out geographically across the region or city, but coordinated centrally.
  - across regions.
    In these virtual systems (“collaborative systems”), the distribution of diagnostic services crosses regional barriers. For example, for rare cancers, diagnostic expertise may be found in only a few locations in the province. Similarly, some procedures may require the use of equipment or technologies readily available in one region but not in another.

Individual regional cancer programs, in collaboration with the local health integration networks, will be responsible for determining the most appropriate organization of the assessment systems. No currently available evidence indicates the population-based volumes required to support any particular model, but it is important to recognize that high-quality diagnostic care is not defined by having a dap for every disease site in every region. Indeed, for rare cancers (for example, head-and-neck cancers or sarcoma), efforts to enhance the current provincial systems of diagnostic and treatment services in a few centres is a more desirable quality goal than is the provision of such services in multiple regions. In contrast, regions should have local mechanisms to deal with the rapid diagnosis of high-volume cancers (for example, lung, breast, colorectal, prostate).

When developing a business case for a specific dap model, the following elements should be considered to justify the choice of model:

- How current diagnostic systems (that is, including the organization of staff, equipment, processes, and so on) within a region can be restructured and redesigned to improve access and quality.
- Volume–outcome literature for specific diagnostic procedures.
- Cost-effectiveness and clinical efficiencies of competing models.
- Population-based estimates of disease incidence and prevalence for each tumour type.

Regardless of the model chosen, meeting common standards for centralized access, scope of activity, team criteria, linkages and collaborations, and performance indicators is required.

- Regional Centralized Access to daps
  A simple and efficient access strategy is a key mechanism for improving the health care experience of the patient and the quality of diagnostic care. Therefore, regardless of the model chosen, a coordinated, centralized, single-point-of-entry, central access system (cas), is an essential element of the dap.

Variation in entry systems may be expected across regions: for example, low and mid-size populations are more likely to be able to support a single-entry cas; a large population region may require a different approach. High-quality diagnostic care can be achieved only by having coordinated points of entry, particularly for the diagnostic work-up of suspected similar cancers, and by implementing systematic referral protocols that supersede existing patterns of referral where quality and access improvements can be made. A cas should be designed explicitly to reduce variations in demand or wait times across the region.

The cas will be responsible for ensuring that eligible patients are brought into a dap and that the diagnostic plans for patients are developed and communicated to the patients, referring physicians, other primary care providers, and local multidisciplinary care conference (mcc) coordinators, using regional and provincial templates. The patient version of the diagnostic plan will include the appointment schedule of all procedures, descriptions of each procedure, and the preparatory activities (if appropriate) for each procedure. The cas will be responsible for communicating the patient version of this plan to the patient by the most appropriate method (telephone, mail, e-mail, Internet). The clinician version will include the appointment schedule of all procedures booked for the patient, and the mcc version will include information about the patient and the appointment schedule of all procedures.

- Entry Points to the cas
  Access to the cas will typically be from a variety of entry points, such as
  - primary care providers or specialists.
    Patients who meet specific cas referral criteria (see “Guidelines and Standards”) will be referred.
  - screening programs.
    Screening programs such as the Ontario Breast Screening Program, the provincial Colorectal Screening Program, and the Ontario Cervical Screening Program will refer to the cas patients who meet specific criteria according to appropriate protocols.
  - self-referral.
    Given the significant proportion of the public who have no access to primary care providers, a system for patient self-referral may be necessary.
    Appropriate pre-screening, following cas protocols, by a qualified clinical coordinator will be required if self-referral is part of the dap. In these instances, the dap should ensure that appropriate primary care services are available to support ongoing care, which may include the development of formal linkages between the dap and primary care networks or family practice teams. Where that is not possible, the dap may need to ensure that these services are provided within the dap itself. The ability of these groups to enter into the dap cas must be demonstrated.
- Operational Features of the cas
  Several operational features are essential elements of a cas:
  - Entry to the cas
    Each dap will determine the most appropriate modality of entry to its cas (telephone, Internet, fax). However, common across all entry strategies for all prospective patients will be the application of referral and triage criteria requirements at the intake point.
  - Fast-access booking
    Protected booking slots must be accessible to the dap for specific diagnostic procedures and clinic appointments with specialists. This approach will distribute patient cases more evenly, facilitate patient flow, and reduce wait times.
  - Priority-booking system
    Triage should be performed by the cas before the first visit to the dap, and an urgent referral mechanism must be implemented for all daps.
  - Open-access booking
    Access to booking for specific diagnostic procedures must be open to all clinicians who adhere to predefined referral criteria and diagnostic assessment protocols (see “Standards and Guidelines”)

### SCOPE OF CANCER DIAGNOSTIC ACTIVITY WITHIN A DAP

Each dap will provide the spectrum of clinical diagnostic and supportive care services for the tumour type or types that fall under the mandate of the program. Appropriate equipment, technologies, and expertise will be required to meet the scope of the diagnostic activities for each assessment unit. Where necessary diagnostic or supportive services are not available, linkages to those necessary services will need to be established to eliminate any gaps in care. The spectrum of diagnostic work-up must be tailored to the specific tumour type, but may include any or all of these services:

- physical examination,
- imaging tests (radiography, computed tomography, magnetic resonance imaging, positron-emission tomography, ultrasonography),
- diagnostic procedures (for example, ultrasound-guided needle biopsy),
- surgical consultation,
- tumour-specific surgical procedures,
- pathology analyses, and
- reporting.

In addition, supportive care services that may be needed include education, psychosocial support, dietetics, genetic counselling, or other types of supportive care.

### CANCER DIAGNOSTIC ASSESSMENT TEAM CRITERIA

It is recommended that assessment services within each dap be composed of a dedicated multidisciplinary team, with each member having explicit roles, responsibilities, and accountabilities. Specialists (for example, gastroenterologists, respirologists) and surgeons will take the clinical lead in the diagnostic processes, with the assessment coordinators serving as primary communication leads. There will be common team elements across the assessment programs, and disease-specific specialists will be required for each dap.

### CANCER DAPS LINKAGES AND COLLABORATIONS

Linkages, collaborations, and communication strategies will vary across the daps. To facilitate patient access, each dap should have formalized bi-directional linkages with primary care providers, other related family health teams or services (including psychosocial support), and any related networks and organizations. Each region will have to develop its own system to fit the specific needs of the region and the various tumour types. There will, however, be some core elements that should be common across all models of diagnostic assessment services.

#### Assessment Coordinator

With the assessment coordinator acting as the main source for information exchange, the assessment programs will establish formal linkages, collaborations, or communication strategies with key stakeholders, including patients entering the cancer diagnostic assessment system, cancer screening programs (where applicable), primary care providers (including family and general practitioners and primary care nurse practitioners), other referral systems, multidisciplinary case conference teams, and related specialists and supportive care services.

#### Primary Care Provider

Formal linkages with primary care providers are essential to a successful dap. Primary care providers must be supported with appropriate tools and products (for example, services plans, guidelines) that provide evidence-based recommendations about appropriate criteria for referral of patients to the dap and committed bi-directional communication with the assessment team, beginning at point of entry, through the patient’s work-up until cancer is diagnosed or ruled out, and to the development and implementation of the treatment plan with a definitive diagnosis.

#### MCC Team/Treatment Team

A clearly identified transition protocol for the patient from the dap to the mcc team or treatment team must be established. The protocol must articulate provider accountabilities and the communication strategy for patients and providers.

### CROSS-DAP COLLABORATION

Formal collaborative linkages among the daps are encouraged. The formal documentation of accountabilities among the various entities or individuals and the dap will be needed, as will communication strategies or protocols with clear reporting formats, to ensure common data collection and reporting, especially regarding outcomes reporting. With standardized reporting systems and clear expectations concerning reporting, the focus should be on accountability and on the collection and delivery of data to enable the assessment of quality indicators and other benchmarks.

Each dap will be responsible for developing a unique diagnostic assessment system, but several models that currently exist within Ontario could help to guide that development. For example, in Ottawa, the Ontario Breast Screening Program has documented the development of a Breast Assessment Program that outlines many key features on which to base a coordinated breast cancer diagnostic assessment service.

### INDICATORS OF QUALITY FOR CANCER DAPS

It is recommended that a range of process and clinical indicators of quality be developed, measured, and monitored to evaluate the performance of each dap. These indicators should reflect the specific needs of each region or tumour type, but they should also be standardized to match provincial benchmarks developed by cco or the Government of Ontario. At both levels, fundamental indicators relevant to the daps should be identified to drive the quality agenda at key points. These must include

- time intervals.
  - The time from abnormal screen or primary care referral to entry into the dap.
  - The time from entry into the dap to cancer diagnosis or rule-out.
- clinical outcomes.
  - Upstaging
  - Mortality
- quality of care.
  The percentage of patients receiving the appropriate diagnostic work-up according to evidence-based guidelines, service plans, and protocols
- patient satisfaction.
  Patient satisfaction throughout the cancer diagnostic assessment system (for example, expansion of the Ambulatory Oncology Patient Satisfaction Survey) Other indicators may include, but are not limited to,
  - program efficiency indicators (avoidance of duplication);
  - completeness of cancer-stage reporting at diagnosis;
  - percentage of pathology reports meeting provincial information completeness standards;
  - clinician team functioning and satisfaction;
  - reporting of cancer services integration through the assessment of linkages, collaborations, and communication both within and external to the dap; and
  - impact on regional performance.

### GUIDELINES, STANDARDS, AND SERVICE FRAMEWORKS

To successfully implement a quality agenda dedicated to reducing wait times for diagnostic services and to improve the quality of those services, there is a requirement for recommendations, benchmarks, and targets, including

- guidelines and service frameworks for primary care providers.
  Facilitation by cco is recommended for the development of provincial evidence-based guidelines and service frameworks for primary care providers. A comprehensive knowledge exchange strategy should be developed and promoted for the uptake of the guidelines.
- evidence-based investigative algorithms and guidance documents.
  Facilitation by cco is recommended for the development of provincial evidence-based algorithms that articulate the most effective diagnostic procedures and the appropriate pathways for the work-up for patients suspected to have cancer. These guideline documents should be developed for all major cancer diagnoses and should serve as the foundation for the local and regional diagnostic pathway protocols and algorithms required to support the daps.
- wait-times benchmarks.
  Facilitation by cco is recommended for the development of provincial benchmark targets for various significant intervals within the diagnostic work-up.

### CONCLUSIONS

An essential need is that implementation of the daps be accompanied by a comprehensive evaluation framework. The standards will evolve and be refined over time as a consequence of the new information gained through the learning experience of implementing the daps. Future iterations will focus on the requirements for comprehensive pathway and risk assessment models for all cancer types in the ongoing effort to improve patient outcomes.
